# Toward an Identification of Resources Influencing Habitat Use in a Multi-Specific Context

**DOI:** 10.1371/journal.pone.0029048

**Published:** 2011-12-27

**Authors:** Emmanuelle Richard, Sonia Said, Jean-Luc Hamann, Jean-Michel Gaillard

**Affiliations:** 1 Unité Mixte de Recherche 5558, Biométrie et Biologie Evolutive, Université Claude Bernard Lyon 1, Bâtiment 711, Villeurbanne, France; 2 Office National de la Chasse et de la Faune Sauvage, Centre National d'Etudes et de Recherches Appliquées sur les Cervidés- Sanglier, Paris, France; Texas A&M University, United States of America

## Abstract

Interactions between animal behaviour and the environment are both shaping observed habitat use. Despite the importance of inter-specific interactions on the habitat use performed by individuals, most previous analyses have focused on case studies of single species. By focusing on two sympatric populations of large herbivores with contrasting body size, we went one step beyond by studying variation in home range size and identifying the factors involved in such variation, to define how habitat features such as resource heterogeneity, resource quality, and openness created by hurricane or forest managers, and constraints may influence habitat use at the individual level. We found a large variability among individual's home range size in both species, particularly in summer. Season appeared as the most important factor accounting for observed variation in home range size. Regarding habitat features, we found that (i) the proportion of area damaged by the hurricane was the only habitat component that inversely influenced roe deer home range size, (ii) this habitat type also influenced both diurnal and nocturnal red deer home range sizes, (iii) home range size of red deer during the day was inversely influenced by the biomass of their preferred plants, as were both diurnal and nocturnal core areas of the red deer home range, and (iv) we do not find any effect of resource heterogeneity on home range size in any case. Our results suggest that a particular habitat type (i.e. areas damaged by hurricane) can be used by individuals of sympatric species because it brings both protected and dietary resources. Thus, it is necessary to maintain the openness of these areas and to keep animal density quite low as observed in these hunted populations to limit competition between these sympatric populations of herbivores.

## Introduction

Habitat selection is a hierarchical process describing the capacity of individuals to choose a habitat that brings resources and conditions necessary for survival and reproduction, and is influenced by temporal and spatial environmental variations [1], [2]. This process involves a set of innate and acquired behavioural decisions, and is shaped by the interplay between habitat preferences of individuals and constraints that prevent them to make the best choice. Thus, interactions between animal behaviour and the environment are both shaping observed habitat use. The normal area that an animal uses to carry out the activities of securing food, mating and caring for young corresponds to the home range [3]. By studying variation in home range size and identifying the factors involved in such variation, we can identify how habitat and constraint influence individual's habitat use.

The home range size strongly depends on energetic needs of individuals [4], [5] that could differ from one species to another, but also from one individual to another according to sex, age and body mass [5]–[8]. The home range size could also change over time according to individual condition (e.g., reproduction status or amount of body reserves), depends on both the landscape global structure and constraints. The landscape global structure has been shown to influence home range size through changes of spatial heterogeneity [9], [10], biomass concentration [11], proximity to cover [12], [13], availability of safe places and of dietary resources [14], [15], wood dispersion [16], number of habitat patches [17], and edge density [18].

Among constraints, population density [9], [14], social interactions [16], intra-specific competition [19], snow accumulation [15], [20], [21], rain and temperature [22], [23], anthropogenic disturbance [6], [24], and topography [9], [25] have all been reported to influence both animal mobility and accessibility to resources. For example, [23] showed an effect of weather on home range size at two different temporal scales. At a short time scale (i.e., daytime) climate modifies animal mobility, whereas at a larger temporal scale (i.e., season) climate impacts the amount and quality of resources available for herbivores. Among constraints, inter-specific competition is likely to have a strong influence on home range size. Indeed, when individuals from different species live in sympatry resource selection by individuals of one species is expected to be constrained by competitive interactions with individuals of other species. Thus, [26] reported that female mule deer (*Odocoileus hemionus*) shifted habitat use by reducing their use of habitats preferred by cattle and by increasing their use of habitat avoided by cattle. Despite the importance of inter-specific interactions on the habitat use performed by individuals, most previous analyses have focused on case studies of single species, so that our understanding of habitat use in a multi-specific context is currently limited.

We aimed here to assess how home range size of sympatric individuals of roe deer (*Capreolus capreolus*) and red deer (*Cervus elaphus*) varies according to the landscape structure in different seasons. Roe deer have a much lower body mass than red deer (18–32 kg vs. 90–220 kg, respectively), leading individuals of these two deer species to have different energetic needs. Moreover, red deer are mixed feeders (sensu [27]) because they feed on both low (i.e., grasses) and high (i.e., fruits and leaves) digestibility food [28]. On the other hand, roe deer are concentrate selectors (sensu [27]) as their diet mostly includes ligneous and semi-ligneous plant species [28]. As a consequence, red deer are able to consume all resources consumed by roe deer, but the opposite is not true [28]. We first focused on resource heterogeneity and diversity, and its link with home range size of both species. Contrary to red deer, roe deer is an ecotone species [16] that selects for edge within its home range. We then expected a negative relationship to occur between resource heterogeneity and/or diversity and home range size (P1) only for roe deer. Secondly, we expected a negative relationship to occur between resource availability and home range size in both deer species because as more resources are available animals should move less to meet their energetic needs (P2). Lastly, we assessed the consequences of the vegetation openness on home range size. In 1999, the hurricane Lothar hit our study area. Lothar increased the amount of resources available for herbivores [30], [31], so we expected a negative relationship to occur between the home range size of both deer species and the proportion of area hit by Lothar (P3a). We expected the same relationship to occur for increased openness generated by forest management, so that home ranges including forest management should be smaller than home ranges without any forest management (P3b).

## Results

We found a great variability in home range size and core area size of both red deer and roe deer. This variability in size was consistently the most important in summer (see Table 1).

**Table 1:** Variation in home range size for both roe deer and red deer according to seasons.

		Home range size			Core area size		
		Min	Max	CV	Min	Max	CV
Diurnal red deer	Spring	126.87	306.24	0.28	18.98	85.32	0.48
	Summer	89.29	286.87	0.42	18.54	99.91	0.48
	Winter	159.69	600.9	0.35	27.58	122.2	0.37
Nocturnal red deer	Spring	116.53	228.69	0.23	14.88	95.65	0.35
	Summer	98.18	215.23	0.27	15.51	47.74	0.39
	Winter	163.08	373.12	0.23	23.55	56.92	0.33
Roe deer	Spring	19.38	52.35	0.28	3.98	11.08	0.32
	Summer	15.47	89.6	0.47	3.42	14.83	0.41
	Winter	22.36	85.03	0.37	5.18	18.61	0.38

Values of the smallest and the largest home range (95%) and core area (50%) in hectares and coefficient of variations (CV) are provided.

### Landscape heterogeneity (see Table 2 for model selection)

**Table 2:** Model selection for the analysis of the variation in roe deer and red deer home range sizes (including both nocturnal and diurnal home ranges for red deer).

Model	Specific Fisher test	F(Df)pvalue
**1) Home range size**		
***a) Roe deer***		
S+ED+S * ED	S * ED	0.15(2)0.85
S+ED	ED	1.92(1)0.17
S	S	6(2)**0.004**
*Selected model: S*		
***b) Diurnal red deer***		
S+ED+S * ED	S * ED	1.74(2)0.15
S+ED	ED	0.37(1)0.54
S	S	22.02(2)5.65*10^−7^
*Selected model: S*		
***c) Nocturnal red deer***		
S+ED+S * ED	S * ED	0.03(2)0.96
S+ED	ED	0.85(1)0.36
S	S	9.88(2)**0.0004**
*Selected model: S*		
**2) Core area size**		
***a) Roe deer***	S * ED	
S+ED+S * ED	ED	0.42(2)0.66
S+ED	S	1.96(1)0.16
S		6.91(2)**0.002**
*Selected model: S*		
***b) Diurnal red deer***		
S+ED+S * ED	S * ED	0.83(2)0.44
S+ED	ED	2.19(1)0.14
S	S	18.26(2)1.26*10^−6^
*Selected model: S*		
***c) Nocturnal red deer***		
S+ED+S * ED	S * ED	0.46(2)0.63
S+ED	ED	0.33(1)0.56
S	S	5.48(2)**0.007**
*Selected model: S*		

Similar model selection was performed for the variation in the core area of the home range. Predictors included habitat variables that describe the landscape heterogeneity: ED (Edge Density). We took into account also the season (S, three levels: Winter, Spring and Summer). We tested the effect of one variable (Specific Fisher Test column) in the model described in the first column. Statistically significant p-values are in bold.

Roe deer home range size differed among seasons (Winter: 45.8±3.24 Ha, Spring: 31.5±3.62 Ha, Summer: 32.31±3.02 Ha) but was not influenced either by edge density or by the interaction between season and edge density (β_Winter Season * Edge density_: 0.032±0.027, β_Spring Season * Edge density_: 0.019±0.032, β_Summer Season * Edge density_: 0.013±0.02). The selected model thus only included between-season differences and accounted for 86% of the variability observed in the roe deer home range size. Similar results occurred for diurnal and nocturnal red deer home range size. Female red deer have a mean diurnal home range size of 278.4±14.08 Ha in winter, 180.06±16.06 Ha in spring and 156.66±12.69 Ha in summer. However, their diurnal home range size was not influenced either by edge density or by the interaction between season and edge density (β_Winter Season * Edge density_: 0.196±0.216, β_Spring Season * Edge density_: 0.324±0.234, β_Summer Season * Edge density_: −0.217±0.204). Similar results were obtained for the nocturnal red deer home range (β_Winter Season * Edge density_: 0.253±0.399, β_Spring Season * Edge density_: 0.284±0.499, β_Summer Season * Edge density_: 0.143±0.634) that covered 319.4±23.9 Ha in winter, 218.75±28.79 Ha in spring, and 156.66±12.69 Ha in summer. The selected models (only including between-season differences for both nocturnal and diurnal home range size) accounted for 52% and 34%, respectively, of the variability observed in the red deer home range size.

Results obtained using core area of home range size (Kernel 50%) were identical to results reported above for home range size (Kernel 95%, see Table 2). The core area of roe deer home ranges differed among seasons (Winter: 9.21±0.64 Ha, Spring: 6.6±0.7 Ha, Summer: 6.59±0.56 Ha) but was not influenced either by edge density or by the interaction between season and edge density (β_Winter Season * Edge density_: 0.002±0.003, β_Spring Season * Edge density_: 0.00009±0.004, β_Summer Season * Edge density_: 0.004±0.003). The selected model (including only between-season differences) accounted for 16% of the variability observed in the core area of roe deer home range size. The same results occurred both for the diurnal core area of red deer home range (β_Winter Season * Edge density_: −0.043±0.028, β_Spring Season * Edge density_: 0.013±0.035, β_Summer Season * Edge density_: −0.028±0.025) that covered 58.25±3.56 Ha in winter, 36.27±3.79 Ha in spring and 29.53±3.37 Ha in summer, and for the nocturnal core area of red deer home range (β_Winter Season * Edge density_: −0.021±0.032, β_Spring Season * Edge density_: −0.02±0.039, β_Summer Season * Edge density_: 0.045±0.066) that covered 63.49±5.06 Ha in winter, 43.55±5.74 Ha in spring and 41.8±5.06 Ha in summer.

### Resource quality and quantity (see Table 3 for model selection and Table 4 for parameter estimates)

**Table 3:** Selection model procedure of variations in roe deer and red deer home range size (both nocturnal and diurnal home range).

Model	Specific Fisher test	F(Df)pvalue
**1) Home range size**		
***a) Roe deer***		
S+TB+BPP+S * BPP+S * TB+BPP * TB	S * TB	0.09(2)0.90
S+TB+BPP+S * BPP+BPP * TB	S * BPP	2.32(2)0.10
S+TB+BPP+BPP * TB	BPP * TB	1.89(1)0.17
S+TB+BPP	BPP	0.03(1)0.86
S+TB	TB	1.74(1)0.19
S	S	6(2)**0.004**
*Selected model: S*		
***b) Diurnal red deer***		
S+TB+BPP+S * BPP+S * TB+BPP * TB	S * TB	0.45(2)0.63
S+TB+BPP+S * BPP+BPP * TB	S * BPP	3.17(2)0.06
S+TB+BPP+BPP * TB	BPP * TB	0.19(1)0.66
S+TB+BPP	TB	0.02(1)0.87
S+BPP	BPP	4.58(1)**0.039**
S	S	26.48(2)9.9*10^−8^
*Selected model: S+BPP*		
***c) Nocturnal red deer***		
S+TB+BPP+S * BPP+S * TB+BPP * TB	S * BPP	0.55(2)0.58
S+TB+BPP+S * TB+BPP * TB	BPP * TB	0.46(1)0.50
S+TB+BPP+S * TB	S * TB	1.49(1)0.23
S+TB+BPP	TB	0.01(1)0.26
S+BPP	BPP	1.3(1)0.26
S	S	9.88(2)**0.0004**
*Selected model: S*		
**2)Core area size**		
***a) Roe deer***		
S+TB+BPP+S * BPP+S * TB+BPP * TB	S * BPP	0.03(2)0.97
S+TB+BPP+S * TB+BPP * TB	S * TB	0.39(2)0.67
S+TB+BPP+S * TB	BPP * TB	0.47(1)0.49
S+TB+BPP	TB	0.04(1)0.84
S+BPP	BPP	4.02(1)**0.049**
S+BPP	S	9.06(2)**0.004**
*Selected model: S+BPP*		
***b) Diurnal red deer***		
S+TB+BPP+S * BPP+S * TB+BPP * TB	S * BPP	0.01(2)0.98
S+TB+BPP+S * TB+BPP * TB	S * TB	0.55(2)0.58
S+TB+BPP+BPP * TB	BPP * TB	0.71(1)0.40
S+TB+BPP	TB	0.47(1)0.49
S+BPP	BPP	7.86(1)**0.007**
S+BPP	S	21.78(2)1.8*10^−7^
*Selected model: S+BPP*		
***c) Nocturnal red deer***		
S+TB+BPP+S * BPP+S * TB+BPP * TB	BPP * TB	4.10^−4^(1)0.98
S+TB+BPP+S * BPP+S * TB	S * BPP	0.05(2)0.94
S+TB+BPP+S * TB	S * TB	1.01(2)0.37
S+TB+BPP	TB	0.06(1)0.80
S+BPP	BPP	15.79(1)**0.0002**
S+BPP	S	12.58(2)4.57*10^−5^
*Selected model: S+BPP*		

Selection procedure was also applied on the core area of the home range. Predictors included habitat variables that describe the quality and quantity of resources: TB (Total biomass) and BPP (the biomass of preferred plants). We took into account also the season (S, three levels: Winter, Spring and Summer). We tested the effect of one variable (Specific Fisher Test column) in the model described in the first column. Statistically significant p-values are in bold.

**Table 4:** Parameter estimates and standard errors under the full model.

	Roe deer		Diurnal red deer		Nocturnal red deer	
	95	50	95	50	95	50
(Intercept)	44.36±7.4	11.43±1.3	341.02±43.2	68.43±6.2	535.4±106.8	92.09±12.8
S _Su_	35.27±4.9	6.44±0.9	125.36±50.8	34.88±8.2	220.2±96.7	48.76±11.4
S _Sp_	30.15±5.9	6.43±1.0	156.74±6.9	44.18±6.9	287.22±83.2	53.79±10.5
TB	0.77±2.7	−0.18±0.2	−22.08±31.1	−2.49±2.1	−123.25±69.9	−6.01±9.1
BPP	3.5±3.0	−0.81±0.6	3.25±28.3	−3.9±1.9	−40.26±37.6	−14.25±10.0
S _Su_* TB	−0.39±0.8	−0.02±0.1	7.88±11.9	−0.54±0.9	2.75±34.9	0.54±4.6
S _Sp_* TB	−0.16±0.9	0.05±0.1	6.33±12.7	−0.99±1.5	3.71±33.2	−0.04±3.6
S _Su_* BPP	−0.11±7.4	−0.91±2.9	67.74±47.2	−3.81±4.6	−166.56±129.4	−11.65±11.3
S _Sp_* BPP	10.64±7.2	−1.85±4.4	56.79±51.4	−4.31±5.0	−142.92±143.5	−15.79±14.4
TB * BPP	−0.9±0.6	0.04±0.5	−12±6.2	0.23±0.5	12.12±11.9	−0.05±2.8

The model includes the effect of season (S; _Su_ for summer and _Sp_ for spring), total biomass (TB), the biomass of preferred plants (BPP) and all double interaction (S*TB, S*BPP, TB*BPP) on home range (95) and core area size (50).

The best model accounted for 14% of the variability observed in roe deer home range size and included between-season differences but no effect of the total biomass, of the biomass of preferred plants, or of any interaction between season and total biomass, between season and the biomass of preferred plants, and between total biomass and the biomass of preferred plants. The same results were found for nocturnal red deer home range size, with the best model accounting for 33% of the observed variability. Results were, however, different for diurnal red deer home range for which the best model (accounting for 46% of the observed variation of size) included the biomass of preferred plants (slope of −19.7±9.2 g/m^2^/Ha) in addition to among-season differences.

The variability in roe deer core area was only influenced by seasonal differences (12% of observed variation accounted for). There was no effect of total biomass, of the biomass of preferred plants, or of any interaction between season and total biomass, between season and the biomass of preferred plants and between total biomass and the biomass of preferred plants. On the other hand, for both nocturnal and diurnal core areas of red deer, seasonal differences (*diurnal core area:* 40% of the variability accounted for; *nocturnal core area*: 15% of the variability accounted for) and the biomass of preferred plant species (*diurnal core area*: slope of −3.2±1.07 g/m^2^/Ha, 6% of the variability accounted for; *nocturnal core area*: slope of −16.33±4.1 g/m^2^/Ha., 22% of the variability accounted for) were retained as structuring factors. There were no effect of total biomass and of any interaction between season and total biomass, between season and the biomass of preferred plants, and between total biomass and the biomass of preferred plants on both the diurnal and nocturnal core areas.

### Influence of the hurricane Lothar and of forest management (see Table 5 for model selection and Table 6 for parameter estimates)

**Table 5:** Selection model procedure of variations in roe deer home range size and red deer home range size (both nocturnal and diurnal home range).

Model	Specific Fisher test	F(Df)pvalue
**1) Home range size**		
***a) Roe deer***		
S+H^2^+FM+S * H^2^+S * FM	S * H^2^	0.30(4)0.87
S+H^2^+FM+S * FM	S * FM	0.06(2)0.93
S+H^2^+FM	FM	2.35(1)0.13
S+H^2^	H^2^	8.88(2)**0.0004**
S+H^2^	S	6.32(2)**0.003**
*Selected model: S+H^2^*		
***b) Diurnal red deer***		
S+H^2^+FM+S * H^2^	S * H^2^	1.01(4)0.42
S+H^2^+FM	FM	1.98(1)0.16
S+H^2^	H^2^	4.47(2)**0.02**
S	S	22.96(2)8.94*10^−7^
*Selected model: S+H^2^*		
***c) Nocturnal red deer***		
S+H^2^+FM+S * H^2^	S * H^2^	0.59(4)0.66
S+H^2^+FM	FM	2.76(1)0.11
S+H^2^	H^2^	4.79(2)**0.016**
S+H^2^	S	8.35(2)**0.001**
*Selected model: S+H^2^*		
**2)Core area size**		
***a) Roe deer***		
S+H^2^+FM+S * H^2^+S * FM	S * H^2^	0.67(4)0.61
S+H^2^+FM+S * FM	S * FM	0.88(2)0.41
S+H^2^+FM	FM	0.51(1)0.47
S+H^2^	H^2^	2.19(2)0.12
S	S	7.75(2)**0.001**
*Selected model: S*		
***b) Diurnal red deer***		
S+H^2^+FM+S * H^2^+S * FM	S * FM	1.78(2)0.18
S+H^2^+FM+S * H^2^	S * H^2^	1.71(4)0.16
S+H^2^+FM	FM	0.06(1)0.81
S+H^2^	H^2^	0.91(2)0.41
S	S	12.88(2)5.59*10^−5^
*Selected model: S*		
***c) Nocturnal red deer***		
S+H+FM+S * H+S * FM	S * FM	0.64(2)0.52
S+H+FM+S * H	S * H	1.09(4)0.37
S+H+FM	H	0.38(2)0.68
S+FM	FM	4.75(1)**0.034**
S+FM	S	3.46(2)**0.039**
*Selected model: S+H^2^*		

Selection procedure was also applied on the core area of the home range. Predictors included habitat variables that describe the hurricane Lothar (H or H^2^ when we tested a quadratic effect) and presence or not of forest management (FM). We took into account also the season (S, three levels: Winter, Spring and Summer). We tested the effect of one variable (Specific Fisher Test column) in the model described in the first column. Statistically significant p-values are in bold.

**Table 6:** Parameter estimates and standard errors under the full model.

	Roe deer		Diurnal red deer		Nocturnal red deer	
	95 (log)	50 (log)	95	50 (log)	95	50 (log)
(Intercept)	3.54±0.2	2.03±0.1	276.26±13.5	3.53±0.3	321.84±25.8	3.907±0.3
S _Su_	3.39±0.1	1.77±0.1	160.67±12.5	3.429±0.1	189.5±25.1	3.411±0.1
S _Sp_	3.37±0.1	1.86±0.1	176.1±22.4	3.69±0.2	221.32±35.4	3.75±0.3
H	−1.35±0.6	0.11±0.6	−204.3±66.8	−4.19±2.8	−166.63±93.6	−2.139±1.4
H^2^	−1.4±0.6	0.664±0.7	−86.05±85.1	−1.45±3.2	−300.64±204.8	−0.64±2.0
FM_Yes_	0.24±0.2	0.25±0.1	No tested	0.276±0.2	No tested	0.098±0.3
S _Sp_* H	−0.948±0.5	−1.09±0.6	−31.35±86.0	−0.95±0.6	−43.29±160.8	0.011±0.8
S _Su_* H	−1.09±0.7	−0.67±0.7	−144.04±193.4	0.1±0.5	−34.61±212.3	−0.115±0.7
S _Sp_* H^2^	−0.631±0.5	−0.063±0.5	−36.7±94.6	−0.68±0.8	−124.78±141.1	−0.091±0.8
S _Su_* H^2^	−0.71±0.6	−0.92±0.7	−183.35±114.6	−0.51±0.6	−155.23±257.1	−1.156±1.0
S _Sp_* FM_Yes_	0.143±0.1	−0.014±0.2	No tested	−0.2±0.1	No tested	0.401±0.2
S _Su_* FM_Yes_	0.084±0.2	−0.077±0.2	No tested	−0.208±0.2	No tested	−0.027±0.4

The model includes the effect of season (S; _Su_ for summer and _Sp_ for spring), quadratic effect of hurricane (H^2^), the presence of forest management (FM) and all double interaction (S*H^2^, S*FM) on home range (95) and core area size (50). Log indicates the logarithmic transformation on size to meet statistical assumptions. Because no biological meaning, we did not test for a two-way interaction between H and FM.

The best model accounted for 35% of the observed variation in roe deer home range size and included seasonal differences and a quadratic effect of the proportion of area damaged by Lothar (slope of −1.12±0.31 and quadratic term of −0.72±0.31, on a log-scale). However, either the presence of forest management or any interaction between season and the quadratic term of the proportion of area damaged by Lothar or between seasons and the presence of forest management influenced the home range size of roe deer. The same result occurred for diurnal red deer home range size (slope of −100.96±46.98 and quadratic term of −93.58±46.41 for the effect of the proportion of area damaged by Lothar; 60% of the variability accounted for by the best model) and for the nocturnal red deer home range size (slope of −120.07±82.36 and quadratic term of −225.75±84.89 for the effect of the proportion of area damaged by Lothar; 39% of the variability accounted for by the best model). However, we did not test for an effect of the presence of forest management (and thereby for any effect of the interaction of this variable with season) on the diurnal and nocturnal red deer core areas because too few individuals had no forest management in their home range (3 and 4 deer, respectively).

For the roe deer core area, the best model accounted for 17% of the variability and only included between-season differences. Either a quadratic effect of the proportion of area damaged by Lothar, the presence of forest management in the home range, the interaction between season and the quadratic term of the proportion of area damaged by Lothar, or the interaction between season and the presence of forest management did not influence the roe deer core area. The same result occurred for the diurnal core area of the red deer home range (41% of the variability accounted for by the best model). However, for red deer during the night, the best model accounted for 21% of the observed variability of core area size and included additive effects of the season and of the presence of forest management in the core area (+0.2954±0.1355 (on the log scale) in the presence of forest management in the core area of the home range). On the other hand, either the quadratic effect of the proportion of area damaged by Lothar, the interaction between season and the quadratic term of the proportion of area damaged by Lothar, or of the interaction between season and the presence of forest management did not influence the nocturnal core area of the red deer home range size.

## Discussion

Our study contributes to a better understanding of which habitat component influences red deer and roe deer home range size when these two species live in sympatry. Contrary to the expectation, we did not find a negative relationship between landscape heterogeneity (measured as edge density) and roe deer home range size. However, as expected, such a relationship did not occur in red deer (for both night and day ranges and for both home range and core area). The biomass of preferred plants inversely influenced the diurnal home range size of red deer but not its nocturnal home range size. There was also no detectable influence of the biomass of preferred plants on roe deer home range size. When considering the core area, we did not detect any effect of the habitat variables for roe deer (although a weak negative influence of the biomass of preferred plants occurred), but we found that the core area of red deer decreases when the biomass of preferred plants during night and day increases. In addition, we did not find a negative effect of total biomass on home range size, contrary to our expectation. Finally, we pointed out that the proportion of area damaged by the hurricane Lothar was the only habitat component that inversely influenced the roe deer home range size. We found the same patterns of variation for both diurnal and nocturnal red deer home range, as we expected. However, Lothar did not influence the core area of any home range (roe deer, red deer during night, and red deer during day). We did not find any evidence that home ranges are smaller in response to forest management, contrary to our expectation. We found even an inverse relationship as the nocturnal core area of red deer home range increased when forest management took place the year before.

Only a few habitat variables were linked to variation in home range size. Habitat has to be heterogeneous to observe a response of home range size to habitat factors. One of the most important component inducing forest heterogeneity is edge density that is generated by natural (hurricane) or human-made (forest management) openness, roads and buildings. Edges bring abundant and high quality forage, so that their utilization rate by deer should be higher than expected by chance [15], [32]. This should be especially the case for roe deer, which is an ecotone species [16], [18]. Contrary to our expectation, the home range size and the core area did not change according to edge density. However, roe deer with the smallest home ranges had more than 30% of their home range hit by Lothar. Thus, Lothar might have caused edge density to be high during the study period and consequently edge density was not a limiting factor for deer. For red deer, the absence of a relationship between home range size or core area and edge density was expected from the specific feeding tactic of this deer species. Indeed, contrary to roe deer, red deer are intermediate feeders (sensu [27]) that can eat both low and high quality resources.

Resource quantity and quality did not influence home range size of roe deer. [33] did not observe between-female differences in overall quantity of resources, but a negative relationship occurred between home range size and resource quality, suggesting that females are able to compensate the size of their home range to get a certain quantity of biomass available. Contrary to previous studies [13], [18], [33], [34], the availability of resources did not shape home range size of female roe deer in our study area, even in spring-summer season, the period of highest energy expenditure. However, we found a weak trend of the core area of home range size to increase with decreasing biomass of preferred plants, suggesting that females adjust the size of the core area of their home range to the amount of resources they can obtain. Contrary to what was reported on Storsfosna [13] and at Chizé [18], some females in our study were able to compensate almost fully the lower food availability by increasing the core area of home range size (slope of −0.81). We found negative relationship between biomass of preferred plants and home range size of red deer during day. The same pattern occurs for the core area of nocturnal red deer home range but we found no relationship with the overall home range. Thus, our results differ from those reported by [35] who suggested that female red deer look for open areas during the day but for closed areas during the night. Both nocturnal and diurnal home ranges were smaller when there included more than 30% of area damaged by Lothar. This suggests that these areas bring to deer both protection and food resources (particularly preferred resources) when they are in sufficient quantity in home range. Thus, animals do not have to move a lot to find the resources they require. This might explain why the composition of both home range and core area of red deer were almost identical between the night and the day. Observed differences of home range size between night and day probably came from human disturbance as during the day female red deer tend to stay longer in protected areas than at night [36].

The absence of any effect of forest management on home range size could come from our rough measure of forest management that included different resources for female roe deer and red deer. Indeed, tie ridge brings protected areas, whereas other works like tree-cutting brings additional resources with the cost of increased disturbance. Contrary to our expectation, we found that the largest core areas of red deer during night included forest management This might correspond to a confounding effect because most of forest management is performed in areas with low food availability (cutting tree in cluster of tall trees), so that deer living in these areas have larger home ranges. We can thus safely conclude that the effect of forest management on the core area of home range was weak at the best.

Home range size depends on interactions between individual energetic needs and spatial distribution of limited resources across the landscape [37]. Home range size and core area varied among seasons, being larger in winter when resources are scarce. This was observed in many species ([14], [33], [38] in roe deer, [9] in elk *Cervus canadensis*, [17] in sika deer *Cervus nippon*, [39] in caribou *Rangifer tarandus*, [40] in white-tailed deer *Odocoileus virginianus*). However, the reverse is observed in other studies where a smaller home range is reported in winter ([20], [35], [41] in white-tailed deer, [42] in izard *Rupicapra pyrenaica*). These results are observed in animals living in areas where snow accumulation is too important during winter and where moving is costlier than staying in [35], [41], [43]. In addition, we did not find any season-specific relationship between habitat component and size of the home range or core area, indicating a unique response of deer to variation in food resources. This might indicate that resource availability was good enough in the reserve, leading to a positive balance between energetic needs and resources. [44] claimed that a given individual should not select its habitat according to a given feature independently of the others, but should rather select a combination of features. The global structure the landscape is shaped by a large set of general factors, so that identifying which one is the most critical for animals is far from being an easy task [24]. Thus factors other than food like individual characteristic could explain observed variation in home range size and the large variability between individuals. For example, previous studies have shown, as a result of increased experience and/or knowledge of the habitat, a decreasing of home range size with age [33]. Inversely, [45] reported increasing home range size with age in male moose. Home range size can also vary according to body size [46]. As the observed variability in home range size was bigger in summer for both deer species, we can suggest that the presence of hider fawns could constraint females to limit their movement. At the same time, summer is the rutting period of roe deer and previous studies have reported that some but not all females make breeding excursions during a few days, leading to a marked increase of their summer home range [47]. In addition to individual characteristic, factors like social network, predation pressure, or human disturbance, might be major determinants of home range size in many species. For instance, [48] have reported an increase of deer home range size in response to an increase of the intensity of grazing by cattle. In the same way, other mammals like red foxes (*Vulpes vulpes*) restricted their range when they are sympatric with competing coyotes [49]. Our results on roe deer differ from those reported in previous studies of the same species [18], [33] probably because of among-site differences in environmental conditions. Roe deer was the only deer species present in the forest in those previous studies. The habitat use by roe deer and red deer in our study area was likely also influenced by the presence of the other species. Red deer are able to consume all resources consumed by the roe deer, but the opposite is not true [29], thus, the absence of relationship between roe deer home range size and biomass of preferred plants (contrary to [11], [33]) could be explained by the presence of red deer. A recent study [50] has reported that young roe deer were lighter when the density of red deer was high, suggesting that a competition can occur between these deer species. From the information we got, we cannot really speak in terms of competition because roe deer could have a refuge by consuming tannin-rich plant-species. Contrary to red deer, roe deer have indeed the ability to detoxify tannins present in brambles, which were abundant in areas damaged by hurricane. We hypothesize that roe deer concentrate on resources they are fittest to limit competition with red deer. Thus hypothesis accounts for the negative relationship we found between areas hit by hurricane and roe deer home range size. In addition, [31] showed that female roe deer decreased by two fold their home range size after the hurricane, and [30] showed that Lothar did not impact roe deer population dynamics. A combination between a high hunting pressure on both deer species that kept these populations at low density and a large amount of food resources brought by Lothar was likely to reduce the competition between deer species. We showed that areas damaged by Lothar influenced in the same way both roe deer and red deer home range sizes. Areas damaged by Lothar have thus a key role in the outcome of inter-specific interaction between roe deer and red deer. However, these areas are highly dynamic, as vegetation grows quickly. To keep a low intensity of competition between red deer and roe deer it might be necessary to maintain open areas damaged by Lothar or even to create new openings in the forest to maintain more than 30% of the area of deer home ranges composed by this open habitat type.

## Materials and Methods

### Ethics Statement

All necessary permits were obtained for the described field studies. The La Petite Pierre National Hunting and Wildlife reserve is managed by the Office National de la Chasse et de la Faune Sauvage and the Office National des Forêts; both institutions were part of and approved our research program. A specific accreditation was delivered to the Office National de la Chasse et de la Faune Sauvage for animal captures (accreditation number 2009-014) and all efforts were made to reduce animal's time handling. Concerning plant species, our method was not intrusive and no sample was taken, therefore, no specific permit was required.

### Study area

La Petite Pierre National Hunting and Wildlife Reserve is a 27 km^2^ forest located in northeast France (48.5°N, 7°E), in the Vosges mountains. The mean elevation is 300 m a.s.l. and the climate is continental with oceanic influences, involving cool summers and mild winters (mean January and mean July temperatures are 0.6 and 18.4°C, respectively, data from Météo France, Phalsbourg weather station, 10 km from La Petite Pierre). Normally, the forest vegetation has a rather low nutritional quality for large herbivores like red and roe deer because the soil is made up of sandstone and is thereby not fertile. However, in 1999, the hurricane Lothar destroyed about 20% of the forest and contributed to increase the amount of vegetation available for herbivores. The forest is structured with even-aged tree stands and includes approximately equal proportions of broadleaved (mainly beech *Fagus sylvatica*) and coniferous (mainly silver fir *Abies alba*, Norway spruce *Picea abies*, and Scots pine *Pinus sylvestris* [51]) species. All the three species of ungulates present in the reserve are hunted, with an average of 40 red deer, 50 roe deer and 150 wild boars (*Sus scrofa*) harvested every year.

### Data collection and home range size estimation

Twenty-five different female roe deer and twenty-three different female red deer were captured between 2004 and 2008 in the reserve, using drive netting or traps. They were released with Lotek GPS 3300S (roe deer), GPS 3300L or GPS 4400M (red deer) collars (Lotek Wireless, Ontario, Canada) and monitored with a schedule of one location every four hours, two days a week. We only kept 3D locations with a DOP<10 and 2D locations with a DOP<5 to remove the less accurate locations [52]. To analyse species distribution during periods of low and high resource availability, we distinguished three seasons: spring (May–June), summer (July–August) and winter (November–February). Red deer are highly sensitive to human disturbance [53], but its diet includes graminoides [29] that can be found in open areas, so that red deer are eating mostly during the night. Consequently, red deer have a biphasic activity [20]. To account for this trait we distinguished day from night in the analyses of red deer data. In order to distinguish night location from day locations, we took into account hours of sunset and sunrise defined by Meteo France. A total of 93 and 56 female-season-year for roe deer and red deer, respectively, were included in the analyses. For the few females that were monitored over consecutive years, we included only one year of GPS locations in the analysis.

We estimated home range size for each period using the 95% fixed kernel estimator [54] with h fixed at 70 meters (71.2±34.4) for roe deer and 140 meters (139.3±58.2) for red deer. These h values corresponded to the mean h-ref values of all animals. [55] showed that fixing h at the same values for all home ranges provides a reliable way to standardize the estimate of home range size and thereby provides a better way to compare home range of different size and number of locations. We also estimated the 50% fixed kernel estimator of home range size, which is the common criterion to identify the core area [56].

### Estimation of resources available

#### Landscape heterogeneity

To measure resource heterogeneity we calculated two metrics from a photo-interpretation map including the twenty-three habitat types that can be found in the study area. We used the Fragstats program [57] in the Patch Analysis extension for Arcview [58]. These metrics included the number of patches per hectare within the area used by individuals (one patch being an area of a particular habitat) and the edge density (measured as the edge length per hectare).

#### Resource quality and quantity

We used two measures of dry biomass to assess the quality and quantity of dietary resources. We estimated the dry biomass per m^2^ using sampling based on the number of plant contacts on a 25×25×165 cm structure (see [59] for details on the method). We used a systematic sampling design, with one sampling location set every 100 meters, across all the reserve in May–June of the years 2004 and 2005. We distinguished the average value of the total dry biomass per m^2^ from the average value of the dry biomass of preferred plant species per m^2^. Roe deer preferred plants were identified following [60]'s work and red deer preferred plants included graminoides, *Picea abies*, *Malus sylvestris*, *Salix* sp., *Sambucus racemosa*, *Vacinium myrtillus*, *Fragaria vesca* in spring-summer and graminoides, *Picea abies*, *Hedera helix*, *Rubus* sp., *Rubus idaeus*, *Ribes* sp. in winter (JLH, unpubl. data). For these two measures, we only retained plants at a height of less than 125 cm for roe deer and less than 165 cm for red deer, which correspond to the species-specific maximal height for feeding.

#### The hurricane Lothar and forest management

Using available maps of tree-cutting by foresters and the maps of the forest damage caused by hurricane Lothar, we recorded whether forestry management (i.e., tree-cutting and tie-ridge) occurred the previous year in the home range and we measured the proportion of the home range damaged by Lothar.

### Statistical analyses

We used each home range size (i.e., 95% kernel, night and day pooled for roe deer and night and day separated for red deer) and each core area size (50% kernel) as dependent variables and season (three-level factor: winter, spring and summer) as covariate. In order to test for the influence of landscape heterogeneity on home range size, we included edge density as a covariate and looked for its interaction with season (First set of models). We did not include the number of patches per hectare in the model because of the high correlation of this variable with edge density (r = 0.84). To test the effects of quality and quantity of resources on home range size, we included the average total dry biomass, the average preferred plant biomass, and possible interactions of these variables with season in the model (Second set of models). Finally, to assess the impact of forest openness on home range size, we included the presence of forest management (two-levels factor: yes or no), the proportion of the area damaged by Lothar, as well as possible interactions among these factors and season in the model(Third set of models). We checked for possible non-linearity for the effects of covariates by fitting quadratic terms. In absence of a clear biological meaning, we did not test for an effect of the two-way interaction between the proportion of the area damaged by Lothar and the presence of forest management.

A log-transformation of the dependent variable was applied when the variances were not homogeneous and/or when the model residuals did not fit a normal distribution. We compared models using Fisher test (alpha fixed to 5%). All the analyses were performed using R 2.10.0 [61].
